# FOOD AND SUPPLEMENT COMBINATIONS TO PREVENT OR POSTPONE AGING, MCI, AND ALZHEIMER’S DISEASE

**DOI:** 10.1093/geroni/igac059.2938

**Published:** 2022-12-20

**Authors:** Alec Pruchnicki, Barrie Kavasch, Barrie Sachs, Roseanne Schnoll, Rolf Martin

**Affiliations:** Vista on 5th, New York, New York, United States; Blueberry Study MMT Corporation, Sherman, Connecticut, United States; MMT Corporation, Sherman, Connecticut, United States; Brooklyn College, CUNY, Sherman, Connecticut, United States; MMT Corporation, Sherman, Connecticut, United States

## Abstract

Evidence of many interacting causes of aging has renewed interest in food and nutrient combinations that may blunt the main causes[PMC9184560, PMC8889622] and postpone aging, mild cognitive impairment and Alzheimer’s (“A-MCI-Alz”) in contrast to prior failures[PMC9232186]. We present an updated list of anti-aging/pro-longevity strategies to address limitations of previous food/nutrient combinations. Our list has two unusual features: (i) all interventions should be administered concurrently so no fundamental causes of A-MCI-Alz are without remediation and can inflame and leave smoldering important causal factors, (ii) all interventions should be provided daily or weekly to help avoid re-ignition of key factors driving A-MCI-Alz. These two features can help explain past longevity-trial failures[PMC9310407]: no previous study to our knowledge has included most or all of the following interventions, so untreated causes of A-MCI-Alz likely remained active[PMC9114803]. Our recommended interventions include intermittent-or-continuous calorie restriction or fasting[PMID: 33138875, PMC9351392, PMC7956384], protein- and/or methionine-restriction[PMC9197402, PMC4023024, PMC9197406], consumption of moderately-strong metal chelators (e.g. polyphenols in blueberries[PMC1413581], other berries[PMC8271923], grapes[PMC7096489, PMC6386230], cherries[PMC8745076], fruits with hesperidin/hesperitin [PMC9310407] like oranges, soybeans with genistein [PMC4286753], ginger [PMC5852742], ginseng [PMC9358063, PMC3830124, PMC6874434], green/rooibos/oolong teas[PMC6567241, PMIDs: 33285593, 19996359], coffee[PMC4926853, PMC9145055], chocolate[PMC9287426]), antioxidant-rich foods[PMC7530501], B vitamins including vitamin B5 (pantothenic acid[PMC9232186, PMC8725342, https://doi.org/10.3181%2F00379727-99-24442]) to help metabolize partly-toxic aldehydes[PMC6802361] and improve/lower lactate/pyruvate and NAD+/NADH ratios[PMC7346061], ingesting adequate-to-high amounts of longevity-correlated tyrosine[PMC6681387], cysteine[PMID: 20191258], glutathione[PMC8912885, PMC5005830], niacin, nicotine acid and/or NADH[PMC7346061, PMC6982340], spermidine[PMC8436989], anti-inflammatory NSAIDS[PMC4270464], autophagy inducers[PMC6191153] fiber[PMC8705837] and fluids[PMID: 21585170] to help remove excess metals, toxic metabolites and damaged organelles, while limiting methionine-rich, other pro-aging foods[PMC4023024].

